# Infinite Mixture Chaining: An Efficiency-Based Framework for the Dynamic Construction of Word Meaning

**DOI:** 10.1162/opmi_a_00176

**Published:** 2025-01-04

**Authors:** Lei Yu, Yang Xu

**Affiliations:** Department of Computer Science, University of Toronto, Toronto, Canada; Cognitive Science Program, University of Toronto, Toronto, Canada

**Keywords:** lexicon, lexical evolution, semantic change, word meaning extension, semantic chaining, probabilistic model, cognitive efficiency

## Abstract

The lexicon is an evolving symbolic system that expresses an unbounded set of emerging meanings with a limited vocabulary. As a result, words often extend to new meanings. Decades of research have suggested that word meaning extension is non-arbitrary, and recent work formalizes this process as cognitive models of semantic chaining whereby emerging meanings link to existing ones that are semantically close. Existing approaches have typically focused on a dichotomous formulation of chaining, couched in the exemplar or prototype theories of categorization. However, these accounts yield either memory-intensive or simplistic representations of meaning, while evidence for them is mixed. We propose a unified probabilistic framework, *infinite mixture chaining*, that derives different forms of chaining through the lens of cognitive efficiency. This framework subsumes the existing chaining models as a trade-off between representational accuracy and memory complexity, and it contributes a flexible class of models that supports the dynamic construction of word meaning by automatically forming semantic clusters informed by existing and novel usages. We demonstrate the effectiveness of this framework in reconstructing the historical development of the lexicon across multiple word classes and in different languages, and we also show that it correlates with human judgment of semantic change. Our study offers an efficiency-based view on the cognitive mechanisms of word meaning extension in the evolution of the lexicon.

## INTRODUCTION

**Figure 1.**
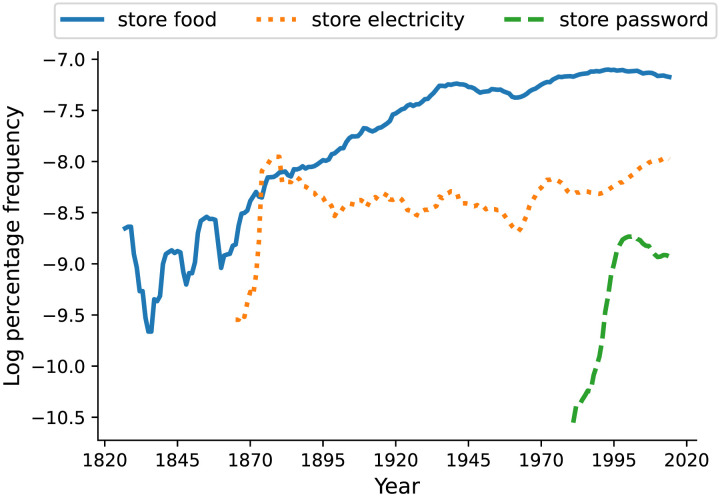
Usage frequencies of the phrases *store food*, *store electricity*, and *store password* in the past two centuries of English. Data were extracted from Syntactic N-grams historical corpus (Goldberg & Orwant, 2013).

A primary function of the lexicon is to support the expression of an unbounded set of emerging meanings with a limited vocabulary. As a result, words often take on new meanings. For example, the word *face* in English originally signifying “body part” was extended later to convey meanings such as “facial expression” and “front surface of an object” (Kay et al., 2015). Similarly, the word *store* took on a variety of novel noun arguments including *food*, *electricity*, and *password* over the past centuries, as illustrated in Figure 1. Word meaning extension is a dynamic process in which words acquire new referents and senses over time, and it is a manifestation of language change which results from a functional need for maximizing communicative expressivity under minimum effort (Blank, 1999; Jespersen, 1959). Existing research has suggested that word meaning extension is non-arbitrary and can be explained partly by cognitive models of semantic chaining (Grewal & Xu, 2021; Habibi et al., 2020; Hilpert, 2007; Lakoff, 1987; Malt et al., 1999; Ramiro et al., 2018; Sun et al., 2021; Xu et al., 2016; Yu & Xu, 2021). However, there is no unified account of different cognitive models of chaining. We present a general probabilistic framework that derives different forms of semantic chaining through the lens of cognitive efficiency.

Word meaning extension is a form of word meaning change (also known as semantic change), which is a long-standing topic of interest to scholars in historical linguistics (e.g., Bréal, 1897; Traugott & Dasher, 2001) and cognitive linguistics (e.g., Lakoff, 1987). Recent work in cognitive science suggests that word meaning extension is a dominant strategy for lexicalizing emerging meanings in the historical development of the English lexicon, and this process relies in part on semantic chaining, also abbreviated as chaining (Ramiro et al., 2018). Chaining refers to incremental mechanisms of word meaning extension whereby new meanings link to existing ones of a word when they are proximal in semantic space, therefore forming chain-like semantic structures over time (Hilpert, 2007; Lakoff, 1987; Malt et al., 1999; Perek, 2018). Existing studies have tested this incremental view by developing computational models of chaining that account for the historical meaning extension of container names (Sloman et al., 2001; Xu et al., 2016), numeral classifiers (Habibi et al., 2020), adjectives (Grewal & Xu, 2021), verbs (Yu & Xu, 2021), and slang terms (Sun & Xu, 2022; Sun et al., 2021).

These previous studies of semantic chaining typically formulate the models in the tradition of two psychological theories of categorization based on prototypes and exemplars. The prototype theory postulates that each lexical category is represented by a central prototype (Lakoff, 1987; Reed, 1972; Rosch, 1975) and has influenced subsequent cognitive linguists who view chaining as a mechanism for generating radial categories. Another account is the exemplar theory of categorization, which proposes that each category is represented by its set of exemplars stored in memory, and it has motivated a line of computational models like the Generalized Context Model that are commonly used to explain human categorization (Ashby & Alfonso-Reese, 1995; Nosofsky, 1986).

Two outstanding issues emerge from these previous studies. First, they often focused on a dichotomous comparison and assumed that the prototype and exemplar models of categorization are sufficient to capture the cognitive processes of chaining in word meaning extension. Second, which of these two models better explains empirical data has received mixed views in different lexical semantic domains (see Grewal & Xu, 2021; Habibi et al., 2020; Yu & Xu, 2021, but also Geeraerts, 1997; Sun et al., 2021), and therefore there is no unified understanding for the different forms of chaining.

Here we propose a general approach to modeling the dynamic construction of word meaning. Our framework not only subsumes the different computational models of chaining but also offers a new class of flexible models that goes beyond the existing accounts of chaining to support the automatic construction of word meaning through time. In particular, we formulate word meaning extension as the process where a target set of head words expand their collocation classes to pair with a larger array of arguments over time, a view that is consistent with studies on lexical semantic change (Allan & Robinson, 2012; Hilpert, 2012). We evaluate our framework against large-scale historical data in different word classes and languages, as well as human judgment of lexical semantic change.

### Theoretical Foundation

Our framework builds on the view that word meanings are structured to support efficient communication (Kemp et al., 2018; Zaslavsky et al., 2018), and that accounts of word meaning extension should take cognitive efficiency into consideration (Ramiro et al., 2018; Xu et al., 2020). Here we define cognitive efficiency as a principled criterion for deriving different formal accounts of semantic chaining, which is based on a tension between two competing constraints that trade off against each other: *representational accuracy* and *memory complexity* (or memory load). Representational accuracy refers to the precision at which a model captures the representation of word meaning, particularly how meaning dynamically changes over time. Memory complexity refers to the cost incurred by a model in meaning representation, particularly the number of stored items required to represent the meanings of words.

**Figure 2.**
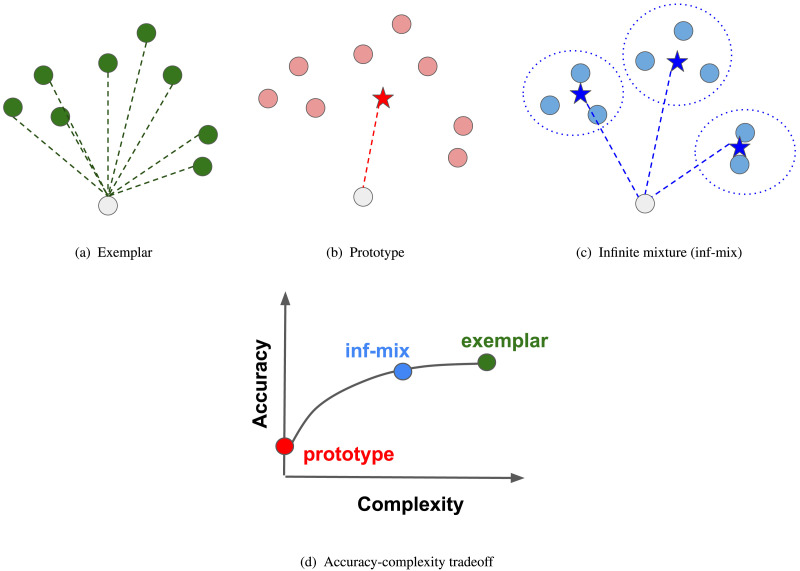
Illustrations of (a)–(c) models of chaining and (d) how they trade off between representational accuracy and memory complexity in the process of word meaning extension. The exemplar model yields high representational precision by linking a novel item (grey dot) to all existing support items (green dots), so it requires high memory complexity. The prototype model requires low memory by linking the novel item to the prototype (red star), but it tends to be less accurate in representation. The infinite mixture model trades off between accuracy and complexity by constructing a semantic space that groups similar items into a sparse set of clusters (dashed circles), and then linking the novel item to the cluster centroids (blue stars).

Under this view, we propose that the existing accounts of chaining including the prototype and exemplar models can be understood as candidates that fall under the two extremes of this trade-off. At one extreme, the exemplar model offers a highly accurate mental representation of a word’s meaning, or lexical category, by storing the past exemplars (i.e., word usages), and it may therefore predict the state of a new item in relation to all the exemplars from memory (see Figure 2a). In this respect, the exemplar model maximizes representational accuracy but at the expense of a high memory load. At the other extreme, the prototype model offers a highly compact representation for a category in terms of a central prototype, and it predicts the state of a new item in relation to that prototype (see Figure 2b). In this respect, the prototype model minimizes memory complexity but at the expense of a simplistic representation, and as a result, the capability of prototype model in predicting or explaining linguistic category extension may be limited compared to exemplar models. The exemplar-prototype dichotomy can thus be interpreted in a unified way as a fundamental tradeoff of cognitive efficiency in word meaning extension: An accurate model tends to demand a high memory load, while a minimum-effort model tends to be more impoverished in representational precision. This efficiency-based framework also offers the possibility to formulate alternative accounts of chaining that go beyond the existing models by near-optimally trading off between the two described constraints of efficiency.

Our proposal is closely related to research in rational human learning and machine learning based on infinite mixtures. Building on this line of work, we model word meaning as an infinite or growing mixture of clusters of usages (see Figure 2c). This modeling scheme can be flexibly adapted to alter the internal structure of a semantic category as it assimilates new items (Alishahi & Stevenson, 2008; Anderson, 1990; Griffiths et al., 2007; Rosseel, 2002; Vanpaemel et al., 2005). In our case, an infinite mixture approach to modeling chaining can potentially capture polysemy (Klein & Murphy, 2001; Li & Joanisse, 2021; Rodd et al., 2012; Tuggy, 1993) and complex structures of word meaning as new items (or usages) emerge over time, therefore moving beyond the exemplar and prototype models which either represent word meanings as a set of independent exemplars or a single prototype. On the other hand, our framework also carries the potential to capture idiosyncratic word usages by storing them as “microclusters” consisting of a single example, therefore offering a “mid-level” structure that lies between schematic and exemplar representations (Dąbrowska, 2004). Similar approaches have been explored in statistical machine learning in the tradition of Dirichlet process (DP) mixture (Allen et al., 2019; Escobar & West, 1995; Ferguson, 1973) which instantiates a trade-off between information loss in model reconstruction of data and complexity in terms of the number of clusters inferred by model (Kulis & Jordan, 2012). Importantly, we demonstrate that the semantic structures inferred by the infinite mixture model can be utilized to predict human judgment of historical semantic change.

## COMPUTATIONAL FRAMEWORK

In the following, we first formulate word meaning extension as a temporal prediction problem under the two constraints of cognitive efficiency described. We then show how several existing classes of chaining models and a new class of models can both be derived from this framework, and we specify the semantic space in which these models are operationalized. Code and data for our work are made available in the following repository: https://osf.io/nsrph/?view_only=9c3f9c0abb9b4679ad5cea7dd6ab3a6e.

### Problem Formulation Under Efficiency Constraints

We define word meaning extension as a temporal prediction problem following the procedures from existing work on semantic chaining that focused on comparing the exemplar and prototype models (Grewal & Xu, 2021; Habibi et al., 2020; Yu & Xu, 2021).

Given a novel emerging item denoted by *n* (e.g., this item can be a new concept that emerges over time such as *password*), we want to predict which existing words in the vocabulary can be extended and chosen to describe it. As a concrete example, we first describe our framework in the case of predicting verb meaning extension, and we show later how it can be applied broadly to other word classes not restricted to verbs.

We cast the problem of word meaning extension as probabilistic inference for predicting novel compositional usages of existing verbs and novel noun arguments. Specifically, given a novel noun *n* such as *password*, we ask which verbs *w* can be taken as its syntactic predicate to form previously unattested compositions that extend the meaning space of *w*, e.g., *store*: “to store food” → “to store password”. We therefore call the word *w* as the *head word* (or *head*), and the nouns that form phrases with *w* the *arguments* of the head. Since a head can take noun arguments under different syntactic roles (e.g., English verbs can take nouns as direct objects, and English adjectives serve as the modifiers of their noun arguments), we also constrain the syntactic relations *r* in predicting novel phrases. Formally, we consider a head-relation pair (*w*, *r*) (e.g., *w* = *store*, *r* = *direct object*) as a time-varying category denoted by ${𝒮}_{w,r}^{\left(t\right)}$ that consists of all existing syntactic noun arguments under relation *r* up to time *t*. The temporal inference problem is then equivalent to predicting the probability of any query noun *n*_*q*_ to emerge in that category at a future time. We focus on predicting pairings of existing heads with query nouns that have not yet appeared as arguments for given heads – for instance, the category “*store (direct object)*” may have been attested include the noun *food* up to time *t*, and can be predicted by a model to extend toward predicating new technological terms such as *information* or *password* later.

Formally, given an emergent query noun *n*_*q*_ at time *t*, and a list of head word forms *w* with existing noun arguments ${𝒮}_{w,r}^{\left(t\right)}$ up to *t*, our framework models the process of semantic chaining in the **head prediction problem** – i.e., inferring which heads will be appropriate predicates for *n*_*q*_ under syntactic relation *r* at time *t* + Δ, where Δ is an incremental time step. The probability of a head *w* being a predicate of *n*_*q*_ via relation *r* is defined as follows:

$$p{\left(w,r|n_{q}\right)}^{t+\Delta }=p\left(n_{q}|{𝒮}_{w,r}^{t}\right)\propto \text{sim}\left(n_{q},{𝒮}_{w,r}^{t}\right)$$ (1)

Our framework can be applied similarly to the **argument prediction problem** asking which novel query nouns will most likely emerge into a head’s referential range in the near future. We achieve this by modeling the conditional probability *p*(*n*_*q*_∣*w*, *r*) of *n*_*q*_ being added as a new syntactic argument of *w*. We take a probabilistic Bayesian approach by computing *p*(*n*_*q*_∣*w*, *r*) as a posterior distribution with a frequency-based prior over heads:

$$p{\left(n_{q}|w,r\right)}^{t+\Delta }\propto p_{0}{\left(w,r\right)}^{t}p{\left(w,r|n_{q}\right)}^{t+\Delta }\propto N{\left(w,r\right)}^{t}\text{sim}\left(n_{q},{𝒮}_{w,r}^{t}\right)$$ (2)

where *N*(*w*, *r*)^*t*^ is the observed frequency count of *w* being a syntactic head of some arguments via relation *r* up to time *t*.

Importantly, sim(*n*_*q*_, ${𝒮}_{w,r}^{t}$) in both of the above equations is a yet-to-be-specified function (i.e., implementing different ways of chaining) that measures the semantic similarity between the query noun and current meaning of the head-relation category 𝒮_*w*,*r*_ at time *t*. To compute this similarity, we quantify the semantic proximity between *n*_*q*_ and the existing set of noun arguments of 𝒮_*w*,*r*_ (i.e., category exemplars). We refer to this set of nouns as the *support set* (denoted by *n*_*s*_ ∈ 𝒮_*w*,*r*_). We assume that the semantic similarity between a query noun and a support set can be captured by the semantic distances between the query and a set of cluster centroids inferred among the support nouns which we denote as 𝓜_*w*,*r*_.

$$\text{sim}\left(n_{q},{𝒮}_{w,r}^{t}\right)=\text{sim}\left(n_{q},{𝓜}_{w,r}^{t}\right)=\text{sim}\left(n_{q},{\left\{\mu_{w,r,k}^{t}\right\}}_{k=1}^{K_{w,r}^{t}}\right)$$ (3)

Here ${𝓜}_{w,r}^{t}=\left\{\mu_{w,r,1}^{t},\mu_{w,r,2}^{t},\ldots \right\}$ is a set of $K_{w,r}^{t}$ cluster centroids for support set ${𝒮}_{w,r}^{t}$. In the next section, we show that exemplar chaining is equivalent to the case where each support noun (or exemplar) forms its own cluster; prototype chaining is the case where all support nouns are represented as a single cluster; and infinite mixture chaining sits in between these two extremes.

We quantify every noun *n* at a given time using distributed semantic representation *ϕ*(*n*)^*t*^ in a high dimensional space that changes over time (details specified in the later section on diachronic semantic space). Following the psychological literature (Nosofsky, 1986), we define semantic similarity as the mean negative exponential Euclidean distance between the query noun and the cluster centroids of a word-relation category:

$$\text{sim}\left(n_{q},{𝓜}_{w,r}^{t}\right)=\frac{1}{K_{w,r}^{t}}\sum_{k=1}^{K_{w,r}^{t}}\exp\left(-\frac{∥\phi {\left(n_{q}\right)}^{t}-\mu_{w,r,k}^{t}{∥}^{2}}{\beta }\right)$$ (4)

where we follow the Generalized Context Model and its variants (Kruschke, 2008; Maddox & Ashby, 1993; Nosofsky, 1986) by adding a sensitivity parameter *β* that controls the rate at which similarity decreases with semantic distance. We allow the number of cluster centroids to flexibly vary over time (as a head word encounters new nouns), which is inferred and updated based on the internal semantic structure of a head-relation category instantiated in terms of its support nouns. In particular, the semantic clusters inferred within a category are expected to optimize the following trade-off between two constraints of efficiency, following work on infinite mixtures from machine learning (Kulis & Jordan, 2012):

$${𝓜}_{v,r}^{t}={\text{argmin}}_{𝓜}\sum_{k}^{K_{v,r}^{t}}\sum_{n_{s}\in S_{v,r}^{t}}∥\phi {\left(n_{s}\right)}^{t}-\mu_{k}^{t}{∥}^{2}+\lambda K_{v,r}^{t}$$ (5)

The first term on the right of Equation 5 is known as the information loss, which quantifies how accurately a set of cluster centroids can represent the full set of support nouns (e.g., in the exemplar model, representational accuracy is near ceiling because each exemplar is in its own cluster). The second term measures the memory complexity for storing cluster centroids (e.g., in the prototype model, memory complexity for a given word is 1, which is the theoretical minimum if we wish to avoid zero representational accuracy). A single parameter *λ* controls the relative weighting between the two constraints. Intuitively, models with higher values of *λ* would favor a more parsimonious approach of chaining by inferring as few clusters as possible (with prototype model at the extreme), while models with smaller values of *λ* would store as many clusters as possible to minimize information loss (with exemplar model at the extreme).

Our formulation of the efficiency tradeoff is also related to the information bottleneck theory of efficient communication, which assumes that word meanings are organized under the tradeoff between reconstruction accuracy and complexity (Tishby et al., 2000; Zaslavsky et al., 2018). However, a crucial distinction here is that our focus is on model inference of newly emerging meanings for individual words rather than the construction of a semantic system where word meanings are held static.

### Classes of Chaining Model

The efficiency formulation in Equation 5 helps derive several classes of chaining model from the literature and anew, and we show that our framework subsumes these classes under a broader spectrum of models.

#### Exemplar-Based Models.

In the case where the tradeoff parameter *λ* → 0, the model ignores the memory constraint and stores every support noun argument *n*_*s*_ as a single cluster to achieve zero information loss. The inf-mix model therefore boils down to the exemplar model of chaining:

$$p{\left(n_{q}|w,r\right)}^{t+\Delta }\propto \frac{1}{|S_{w,r}^{t}|}\sum_{n_{s}\in S_{w,r}^{t}}\exp\left(-\frac{∥\phi {\left(n_{q}\right)}^{t}-\phi {\left(n_{s}\right)}^{t}{∥}^{2}}{\beta }\right)$$ (6)

The literature has also suggested that a variant of the exemplar model, particularly 1-nearest-neighbor (1nn) chaining, has been effective in predicting emergent word senses (Ramiro et al., 2018). If we adjust the inference procedure by considering only one support noun closest to the query noun (in semantic space) instead of all the support nouns, we can easily derive the 1nn chaining model:

$$p{\left(n_{q}|v,r\right)}^{t+\Delta }\propto {\text{argmax}}_{n_{s}\in S_{v,r}^{t}}\exp\left(-\frac{∥\phi {\left(n_{q}\right)}^{t}-\phi {\left(n_{s}\right)}^{t}{∥}^{2}}{\beta }\right)$$ (7)

#### Prototype Model.

If *λ* → ∞, the model yields a minimal memory cost by storing only a single cluster centroid (or the prototype) for each category, and it therefore converges to the prototype model:^1^

$$p{\left(n_{q}|w,r\right)}^{t+\Delta }\propto \exp\left(-\frac{∥\phi {\left(n_{q}\right)}^{t}-\mu_{w,r}^{t}{∥}^{2}}{\beta }\right)$$ (8)

Here $\mu_{w,r}^{t}=\frac{1}{|S_{w,r}^{t}|}\sum_{n_{s}\in S_{w,r}^{t}}$ *ϕ*(*n*_*s*_) is the mean embedding of all nouns in a support set.

#### Infinite Mixture Model (inf-mix).

In the intermediate cases where 0 < *λ* < ∞, the number of clusters lies between 1 and the support set size ∣$S_{w,r}^{t}$∣ and can be inferred using a deterministic algorithm called DP-Means (Kulis & Jordan, 2012). This is a nonparametric variation of the well-known K-means clustering algorithm in unsupervised learning (Hartigan & Wong, 1979). The centroids ${𝓜}_{w,r}^{t}$ would then be the mean vector representation of the support arguments within each cluster. Figure 2 illustrates the different classes of chaining model in the computation of *p*(*n*_*q*_∣*w*, *r*). Theoretically, it can be shown that the infinite mixture chaining model is equivalent to the asymptotic case of a Dirichlet Process Gaussian Mixture Model (DPGMM) (Görür & Rasmussen, 2010) with the variance parameter of the Gaussian likelihood function shrunk toward 0 (Kulis & Jordan, 2012). However, in a fully Bayesian DPGMM, the mixture centroids *μ*_*k*_ become latent variables and need to be inferred via posterior sampling, which requires storing and repeatedly using all support noun arguments. This is computationally prohibitive for common head-relation classes consisting of hundreds or even thousands of noun arguments. Our framework bypasses these issues of DPGMM and is more computationally efficient.

### Semantic Space

The chaining models described need to be operationalized in a time-varying semantic space so that information about future head-argument phrases should be minimally smuggled into prediction at current time points. We use Word2Vec-based representations commonly used in natural language processing for distributed semantics (Mikolov et al., 2013). Note that word co-occurrence distributions are constantly changing and therefore the semantic space needs to be updated to capture information only up to time *t*. For this reason, we use the 300-d HistWords pre-trained diachronic embeddings (Hamilton et al., 2016), where the embedding for each noun at decade *t* is based solely on its co-occurrence statistics from the current decade, while the future co-occurrences are not embedded. Other studies have explored multimodal representations of word meaning beyond textual data (Brochhagen et al., 2023; De Deyne et al., 2021; Yu & Xu, 2021), which can provide alternative semantic representations.

## DATA

We evaluate our infinite mixture chaining framework on reconstructing historical extension in three classes of words, derived from three separate datasets building on the existing literature of semantic chaining: 1) English verb phrases consisting of head verbs and noun objects (cf. Yu & Xu, 2021), 2) English adjective phrases of head adjectives and modified nouns (cf. Grewal & Xu, 2021), and 3) Chinese numeral classifiers and their measured nouns (cf. Habibi et al., 2020). Table 1 shows sample entries from these datasets which we describe in turn.

**Table 1.** Sample entries from Google Syntactic N-grams including head-relation pairs, support and query nouns, and timestamps.

Decade	Head-relation pair		Support noun	Query noun
	Head word	Syntactic relation		
1900	drive (verb)	direct object	horse, wheel, cart	car, van
1950	work (verb)	prepositional object via *as*	mechanic, carpenter, scientist	astronaut, programmer
1980	healthy (adjective)	modified objec	food, diet, life	vegan, finance
2000	次(cì) (Chinese classifier)	modified object	资助(funding), 就业(employment), 发言(speech)	公投(referendum)

### Historical Data of English Verb Phrases

Building on the study of Yu and Xu (2021), we collected a large dataset of historical head-argument compositions derived from the Google Syntactic N-grams (GSN) English corpus, where the noun argument can be either the direct object of the verb (e.g., store the *password*) or can be an indirect prepositional object (e.g., store *in the computer*). In particular, we collected verb-noun-relation triples (*n*, *v*, *r*)^*t*^ that co-occur in the ENGALL subcorpus of GSN from 1850 to 2000. We focused on working with common usages and pruned rare cases under the following criteria: 1) all noun arguments are extracted from a large vocabulary of words with top-10,000 noun counts (with POS tag as noun) in GSN over the 150-year period; 2) all verbs should have at least 20,000 counts in GSN. To support feasible computations, we consider the top-20 most common syntactic relations in GSN between head verbs and noun arguments. We binned the raw co-occurrence counts by decade Δ = 10. At each decade, we define emerging noun arguments for a given verb-relation category (*v*, *r*) if their number of co-occurrences with (*v*, *r*) up to time *t* falls below a threshold *θ*_*q*_, while the number of co-occurrences with (*v*, *r*) up to time *t* + Δ is above *θ*_*q*_ (i.e., an emergent usage that conventionalizes over time, as opposed to a spontaneous usage). We define support nouns as those that co-occurred with (*v*, *r*) for more than *θ*_*s*_ times before *t*. We found that *θ*_*q*_ = 10 and *θ*_*s*_ = 100 are reasonable choices. This preprocessing pipeline yielded a total of 8,897 verb-relation categories of noun arguments over 15 decades, where each category has at least 1 novel query noun and 10 existing support nouns in each decade.

### Historical Data of English Adjective Phrases

Analogous to verb extension and building on the study of Grewal and Xu (2021), we extracted historical compositions of English adjective modifiers and their noun arguments from the ENGALL subcorpus of GSN from 1850 to 2000. We also pruned rare usages by keeping only the same set of top-10,000 most frequent noun types as in the verb phrase dataset, and removing phrases whose head adjective has less than 20,000 counts in GSN. At each decade, the criteria of deciding novel emergent noun arguments for an adjective is the same as those described in the verb phrase collection pipeline. We finally obtained a total of 2,037 adjective categories of noun arguments over 15 decades, where each category has at least 1 novel query noun and 10 existing support nouns in each decade.

### Historical Data of Chinese Numeral Classifiers

Building on the study of Habibi et al. (2020), we also apply inf-mix chaining to reconstruct meaning extension of linguistic categories beyond English. Specifically, we consider how Chinese numeral classifiers have been applied to modify novel nouns in the 20th century. Chinese classifiers are obligatory grammatical classes that are used between a noun and a numeral term describing its quantity, e.g., one [个(gè)] person or two [份(fèn)] documents. We made use of the data of historical linguistic category extension in Habibi et al. (2020), which includes a comprehensive list of 8,371 (Chinese classifier, noun) pairs over the period 1940–2003. We follow Habibi et al. (2020) by representing each Chinese noun as the pretrained word2vec contemporary embedding of its English translation to prevent information smuggling. Different from Habibi et al. (2020) that predicted novel usages on a yearly basis, we binned the noun-classifier pairs by Δ = 5 based on their time of emergence to ensure that between two consecutive evaluation time points, there is a sufficient number of newly introduced arguments and a set of significantly different inferred semantic centroids by inf-mix models.

## RESULTS

In this section, we first show via a simulation study that inf-mix chaining offers better account for the extension process of categories with various structures. We then demonstrate in three case studies that cognitive efficiency can derive new chaining models that balance the trade-off between predictiveness of new meanings and memory complexity. We finally show that the semantic representations derived from inf-mix chaining is psychologically grounded in that they can capture human judgment of diachronic word meaning change.

### Infinite Mixture Chaining on Simulated Data

We first compare inf-mix and existing models of chaining using simulated data of category extension in a continuous two-dimensional space. To do so, we generate *N* = 500 data points for each of two competing category via Gaussian Mixture models with randomly sampled components. We then present a chaining model with 80% of generated points from each category as the training set to infer category centroids and use the model to predict category labels for the remaining 20% as the test set. We test a series of 50 inf-mix chaining models whose inferred number of semantic centroid ranges from 1 (prototype) up to 500 (exemplar) with a step-size of 10. In each simulation, the number of Gaussian component for each category is randomly sampled between 1 and 10, and the mean of each Gaussian distribution is randomly drawn from a 2-D uniform distribution in [−1, 1] × [−1, 1]. We keep all Gaussian distributions isotropic with a variance of 0.1 in both dimensions.

**Figure 3.**
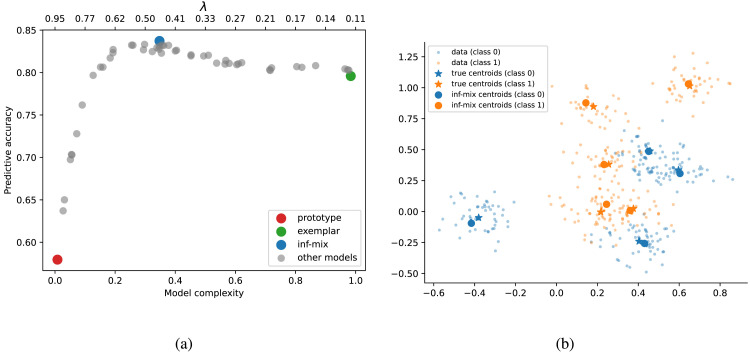
(a) Predictive accuracy and memory complexity (ratio between inferred cluster number and category size) on 2-D simulated data. (b) Example generated data points and inferred cluster centroids by inf-mix for two competing categories with multiple Gaussian mixture components.

Figure 3a illustrates the trade-off between mean model predictive accuracy on test set and normalized model complexity measured by the ratio between the number of inferred clusters and the category size. We found that a range of inf-mix models with moderate complexities best balance the trade-off by offering the highest predictive accuracy while maintaining a relatively low memory cost. In contrast, the exemplar model is less accurate and more memory-intensive, while the prototype model, despite having the lowest memory cost, yields the lowest predictive accuracy that is only slightly above chance. Figure 3b shows the generated data points of two Gaussian mixture categories with 4 and 5 components respectively, together with the inferred cluster centroids by the inf-mix model of highest test accuracy. In this case, the inf-mix model almost perfectly recovers the ground-truth centroids for both categories. These simulation results suggest that for artificial categories with various structures, inf-mix chaining has the potential to offer the most cognitively efficient account of category extension.

### Case Study 1: English Verb-Noun Compositions

We next evaluated empirically different classes of chaining models on both head and argument prediction problems under variation of the trade-off parameter *λ* on emerging verb-noun compositions for the historical period 1850 to 2000. At every decade, for argument prediction problem, we randomly sample for each verb-relation pair (*v*, *r*) 100 candidate noun arguments (with exactly one of them being a ground-truth novel argument *n*_*q*_) from the vocabulary of top-10,000 nouns in GSN, and we then compute the percentage of cases where each chaining model predicts the true *n*_*q*_ over the random noun set as a more appropriate argument. Similarly, for the head prediction problem, we randomly sample for each query noun 100 candidate verb-relation pairs (with exactly one of them being a ground-truth head of the query noun) from the vocabulary of top-5,000 verb-relations in GSN. This procedure allows us to assess the degree to which each class of chaining model can successfully predict novel verb-noun compositions incrementally through time, and how they fare in the accuracy-complexity trade-off. To pursue best model predictability of word meaning extension, we apply stochastic gradient descent to tune the sensitivity parameter *β* in the semantic similarity functions of each chaining model to maximize their average predicted probability *p*(*n*_*q*_∣*w*, *r*)^*t*+Δ^ over all ground-truth (novel noun argument, head word, relation) triples in the dataset.

For infinite mixture models with 0 < *λ* < ∞, we implemented the DP-means clustering algorithm introduced in Kulis and Jordan (2012) to assign a categorical cluster label for every noun within the support set of each verb-relation pair, and take the mean word embeddings of support nouns in each inferred cluster as centroid to compute the likelihood function *p*(*n*_*q*_∣*v*, *r*). Since Euclidean-distance-based clustering methods such as DP-means tend to degenerate on high dimensional data (due to the curse of dimensionality), we instead perform DP-means on a 30-dimensional subspace of the HistWords embeddings projected by principal components analysis (PCA). We found that this reduced subspace preserves well the relative distances between word pairs (explaining over 80% of variance from the original 300-dimensional data) and yields reasonable clustering results. During prediction, we use the full HistWords embeddings by computing centroids using clustering labels computed on the PCA subspace.

We found that when *λ* = 0.24 and *β* = 0.45, the inf-mix model yields most well-defined clustering overall measured by the standard Silhouette score for unsupervised clustering.^2^ We therefore evaluate this model on its predictive accuracy by-decade and aggregate predictive accuracy, along with the other competing models. We also consider two baseline models: a frequency baseline that always favors the noun with the highest usage frequency in GSN up to the decade in question, and a random baseline.

**Figure 4.**
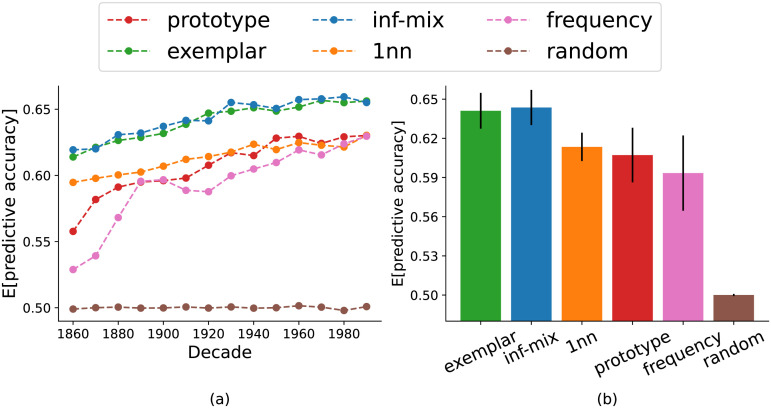
Model accuracy of head verb prediction through time (left panel) and in aggregate (right panel). The infinite mixture model has *λ* = 0.24. Error bars represent the standard deviations of accuracy across decades.

**Figure A1.**
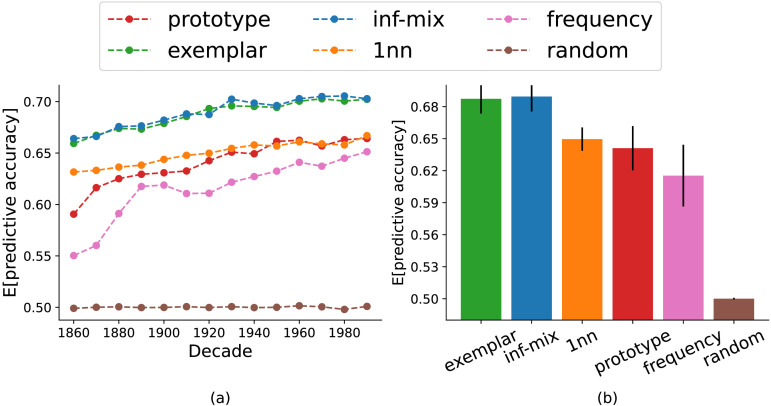
Model accuracy of argument noun prediction for English verbs through time (left panel) and in aggregate (right panel). The infinite mixture model has *λ* = 0.24. Error bars represent the standard deviations of accuracy across decades.

Figure 4 summarizes the results for the main problem of head prediction. We observe that among all the models examined, the infinite mixture and exemplar models yielded near-equivalent predictive accuracy and are superior than the alternatives. The prototype and 1nn models perform better than the two baselines, but they are much worse than the top 2 models. We observed similar result patterns in the argument prediction problem as well (see Figure A1 in Appendix). These initial results show that the infinite mixture model is on par with the exemplar model in predicting historical verb extension, the latter being the better performing model as reported in recent work of chaining (Grewal & Xu, 2021; Habibi et al., 2020; Yu & Xu, 2021).

**Figure 5.**
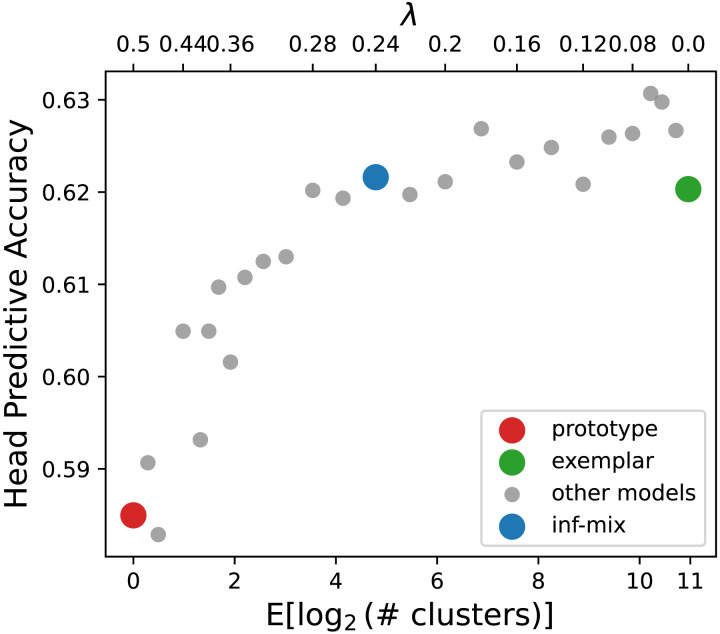
Accuracy of head prediction problem and memory complexity (mean number of clusters per word used in prediction) on English historical verb-noun composition dataset. The gray dots show the spectrum of infinite mixture models under different *λ* values. The left end (red dot) of the x-axis corresponds to the prototype model with *λ* ≥ 0.5. The right end (green dot) of the x-axis corresponds to the exemplar model with *λ* = 0. The blue dot corresponds to the infinite mixture model with inferred optimal *λ* value.

**Figure A2.**
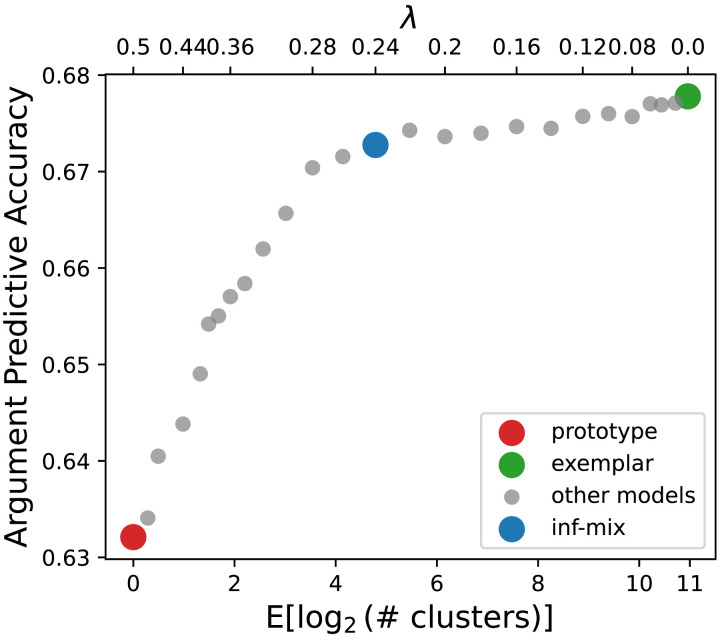
Accuracy of noun argument prediction problem and memory complexity (mean number of clusters per word used in prediction) on English historical verb-noun composition dataset. The gray dots show the spectrum of infinite mixture models under different *λ* values. The left end (red dot) of the x-axis corresponds to the prototype model with *λ* ≥ 0.5. The right end (green dot) of the x-axis corresponds to the exemplar model with *λ* = 0. The blue dot corresponds to the infinite mixture model with inferred optimal *λ* value.

To assess the efficiency of different chaining models, we computed the expected predictive accuracy from the average predictive percentages over all (*v*, *r*, *n*_*q*_) triples in the dataset. We also measured memory complexity by computing the expected number of cluster centroids inferred for every set of support noun arguments at each decade. We focus on comparing the infinite mixture model with the two most representative models of chaining, prototype and exemplar. We also incrementally vary *λ* to assess a large set of other alternative classes of chaining beyond the three target models. Figure 5 shows the result for head verb prediction which indicate that 1) by sweeping *λ* from 0 toward ∞ (in this case *λ* ≥ 0.5 suffices), the predictive accuracy drops only slightly from the exemplar model to the infinite mixture model (*λ* = 0.24) but substantially to the prototype model—this finding confirms with our previous analysis, that the infinite mixture model predicts on par with the exemplar model; and 2) the marginal gain on accuracy of the exemplar model comes at a high cost in memory complexity: compared to the infinite-mixture model, it requires over 2^5^-fold more storage of cluster centroids to achieve a gain of <0.01 in predictive accuracy. We observed a similar trade-off curve for the argument noun prediction problem as well (see Figure A2 in Appendix). Overall, the infinite mixture model achieves a better balance between precision and memory.

**Figure 6.**
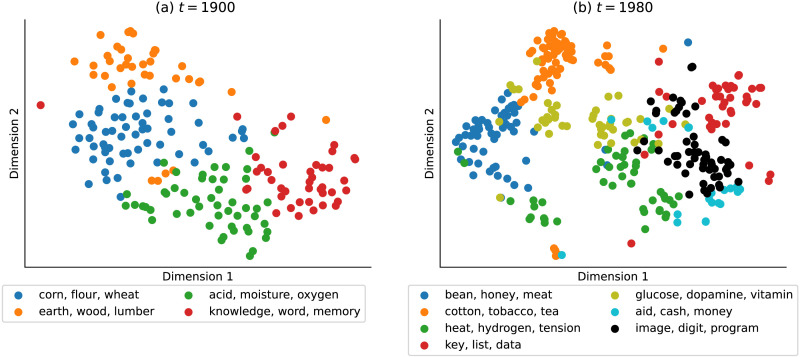
Low-dimensional visualizations of historical meaning extension for the verb frame *store in (noun)* from 1900s (left) to 1980s (right) via t-SNE projection. The dots correspond to word embeddings of noun arguments grouped in clusters inferred by the infinite mixture chaining model. Legends show 3 representative nouns closest to their cluster centroids for each cluster.

We interpret the semantic clusters learned by the infinite mixture model using verb category *store in dobj* as an example. Figure 6 illustrates its meaning space spanned by support nouns in 1900s and 1980s respectively, projected on a 2D plane using the t-distributed Stochastic Neighbor Embedding (van der Maaten & Hinton, 2008). The model identifies 4 clusters of semantically related *store*-able nouns in 1900s and 7 clusters in 1980s, most representative noun arguments for which are shown in the legends of Figure 6. To track how these the meaning clusters change over time, we mark a pair of clusters across the two decades with the same color if they share the highest number of overlapping support arguments. For instance, the cluster with arguments *bean*, *honey*, *meat* in 1980s is colored in blue, since it shares the most support nouns with the *corn*, *flour*, *wheat* cluster at 1900s. The three clusters in 1980s with distinct colors (marked in olive, cyan and black) can be considered as novel senses that the verb category acquired during the 20th century. We found that the infinite mixture model not only infers consistent noun clusters across time by adding semantically related novel nouns to the existing clusters (e.g., assigning words like *key*, *data* to the red cluster denoting abstract concepts related to knowledge and mind), but also detects novel word senses by growing clusters that contain those emerging concepts (e.g., the olive cluster of biology terms, and the black cluster that contains information technology terms).

### Case Study 2: English Adjective-Noun Compositions

Similar to the previous case study on English verb phrases, we evaluated chaining models trade-off parameter *λ* ∈ [0, 0.6] on predicting emerging noun arguments for English adjectives between 1850 and 2000. Each trial of head prediction consists of a true attested adjective and 99 randomly sampled adjectives that are never paired with the target noun, and each trial of argument prediction consists of a true attested argument noun and 99 randomly sampled nouns that are never paired with the target adjective. All chaining models are again evaluated on a PCA-reduced 30-dimensional HistWord historical semantic space, in which we also performed DP-Means clustering for inf-mix models and found that *λ* = 0.14 and *β* = 1.50 yielded the most coherent clustering results (measured by the Silhouette score).

**Figure 7.**
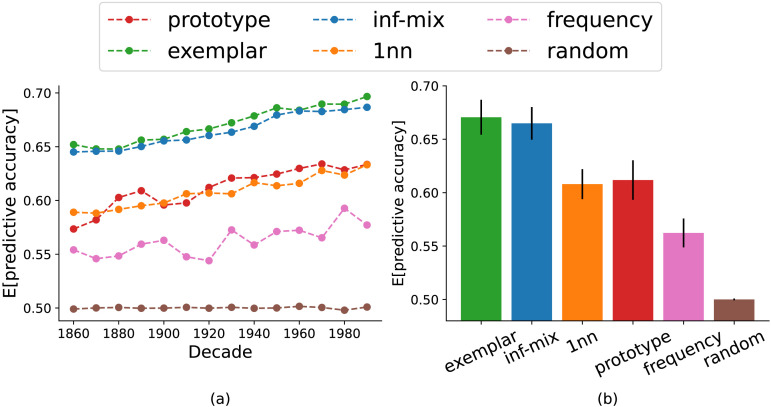
Model accuracy of head adjective prediction through time (left panel) and in aggregate (right panel). The infinite mixture model has *λ* = 0.14. Error bars represent the standard deviations of accuracy across decades.

**Figure 8.**
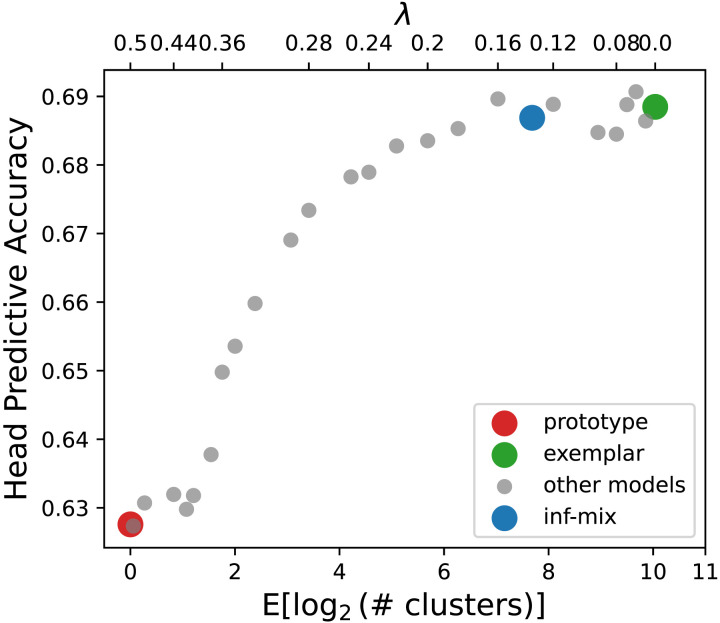
Accuracy of head prediction problem and memory complexity (mean number of clusters per word used in prediction) on English historical adjective-noun composition dataset.

**Figure A3.**
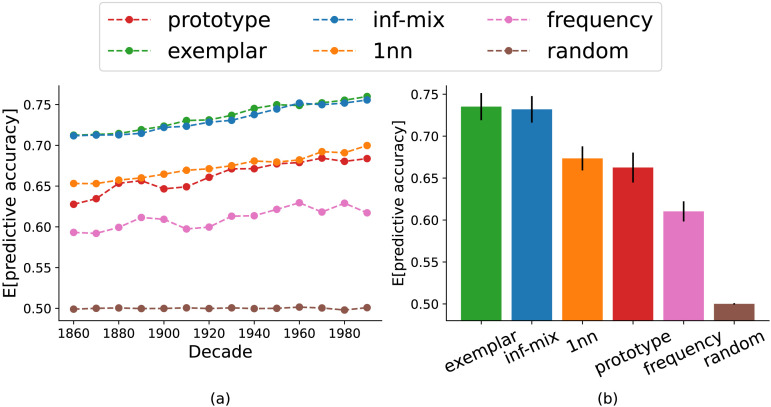
Model accuracy of argument noun prediction for English adjectives through time (left panel) and in aggregate (right panel). The infinite mixture model has *λ* = 0.14. Error bars represent the standard deviations of accuracy across decades.

**Figure A4.**
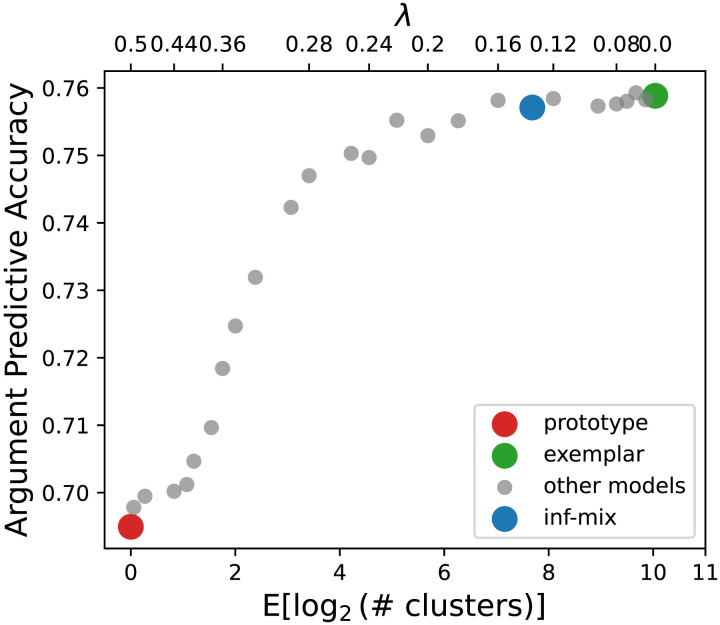
Accuracy of noun argument prediction problem and memory complexity (mean number of clusters per word used in prediction) on English historical adjective-noun composition dataset.

Figure 7 summarizes the predictive accuracy for all chaining models on the main task of head prediction. We again observe that among all the models examined, the infinite mixture and exemplar models yielded near-equivalent predictive accuracy and are superior than the alternatives. Figure 8 summarizes the memory complexity and predictive accuracy on head adjective prediction task for all models. Again, the most coherent infinite mixture model with *λ* = 0.14 and exemplar models yielded nearly identical performance, whereas the latter requires approximately four times more memory space to operate. and as *λ* goes above the critical value of 0.14, the performance of models of decreasing memory cost drops significantly. We observed similar result patterns in the argument prediction problem as well (see Figure A3 and A4 in Appendix). These results conform with our finding in case 1 and suggest that the inf-mix chaining model offers the best trade-off between precision and memory in accounting for historical semantic change of English adjectives.

### Case Study 3: Chinese Classifier-Noun Compositions

**Figure 9.**
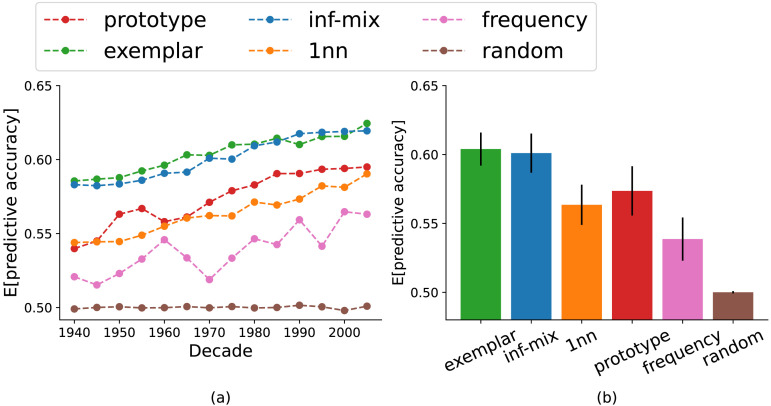
Model accuracy in head classifier prediction through time (left panel) and in aggregate (right panel). The infinite mixture model has *λ* = 3.0. Error bars represent the standard deviations of accuracy across decades.

**Figure 10.**
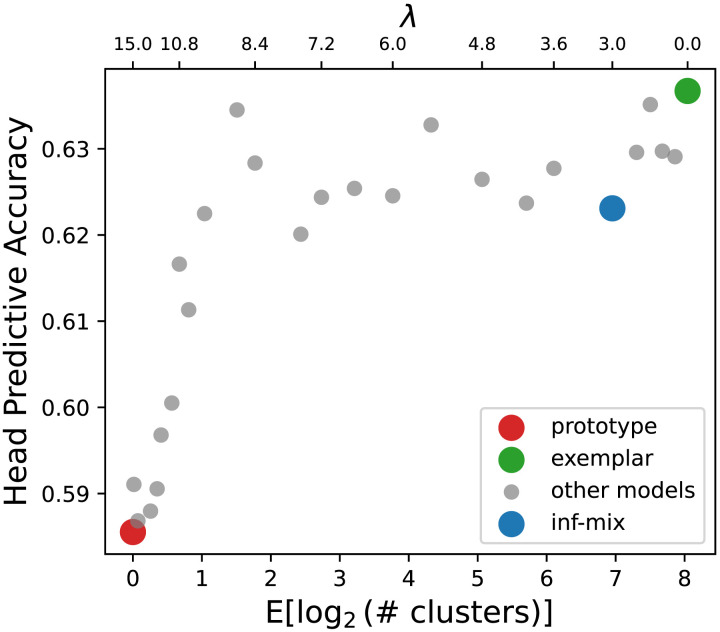
Accuracy of head prediction problem and memory complexity (mean number of clusters per word used in prediction) on Chinese historical noun-classifier composition dataset.

**Figure A5.**
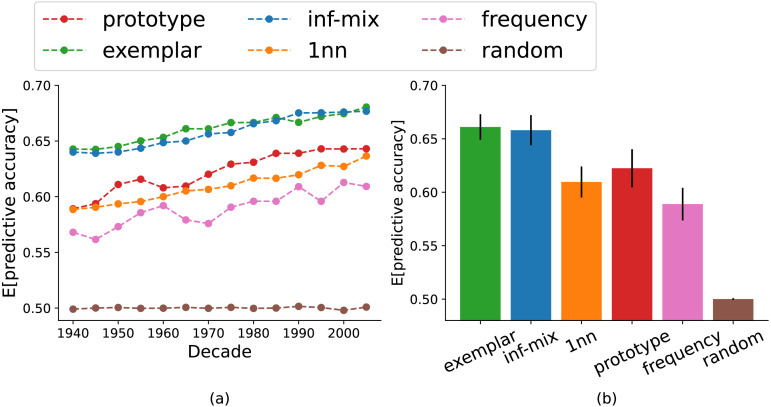
Model accuracy in argument noun prediction for Chinese numeral classifiers through time (left panel) and in aggregate (right panel). The infinite mixture model has *λ* = 3.0. Error bars represent the standard deviations of accuracy across decades.

**Figure A6.**
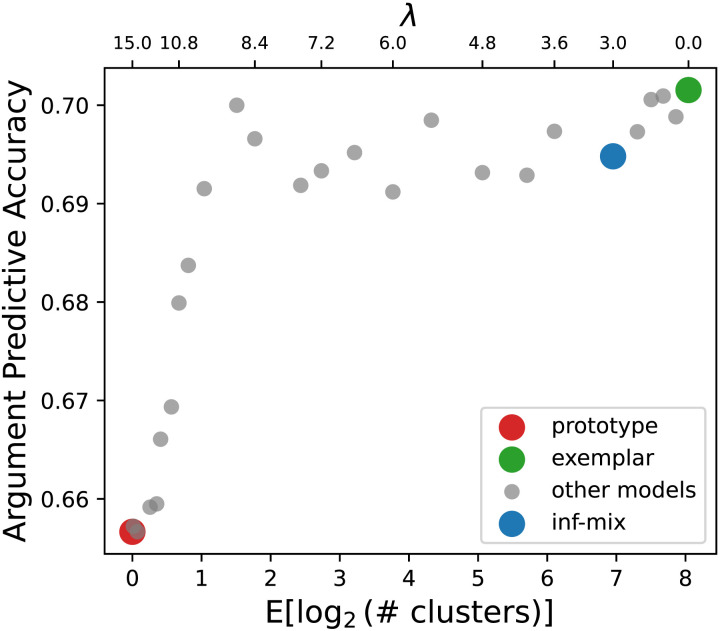
Accuracy of noun argument problem and memory complexity (mean number of clusters per word used in prediction) on Chinese historical noun-classifier composition dataset.

Figure 9 summarizes the expected head prediction problem accuracy over all (classifier, novel noun) pairs in the Chinese numeral classifier dataset for all chaining models. Similar to the previous two case studies, the infinite mixture and exemplar models are on par with each other while outperforming all other models. Figure 10 summarizes the memory complexity and predictive accuracy on the head classifier prediction task. Due to the different geometric properties of the contemporary word2vec embeddings, we found that in this case the inf-mix model converges to the prototype model at a much larger *λ* ≈ 15.0 compared to thresholds in the previous two experiments, and a sensitivity parameter *β* = 0.96. Moreover, we observed that the optimal inf-mix model of the most coherent semantic clusters is more memory-intensive than the previous two optimal inf-mix models (with an optimal *λ* ≈ 3.0), suggesting that the meaning of Chinese classifiers is much more “polysemous” compared to English verbs and adjectives, as the noun arguments of Chinese classifiers cannot be summarizes using a small set of semantic classes. However, despite the more complex nature of classifier meaning, the performance of the inf-mix model again matches the best exemplar model with about 50% less memory requirement. We observed similar result patterns in the argument prediction problem as well (see Figure A5 and A6 in Appendix).

Taken together, the three case studies suggests that inf-mix offers a novel view and extends existing models of word meaning extension, by incorporating cognitive efficiency into semantic chaining to quantify the trade-off between predictive precision and memory complexity.

### Case Study 4: Human Judgment of Lexical Semantic Change

Finally, we perform a quantitative analysis to assess whether the inferred semantic centroids by inf-mix chaining model can be applied to explain people’s judgment of diachronic lexical semantic change (LSC) in English. We choose the subtask 2 of SemEval-2020 Task 1 (Schlechtweg et al., 2020) as our evaluation dataset, where an unsupervised model is asked to rank a set of target words according to their degree of lexical semantic change between corpora *C*_1_ and *C*_2_ from two different time periods. For the case of English, *C*_1_ and *C*_2_ are the subsets of the Clean Corpus of Historical American English (CCOHA) (Alatrash et al., 2020) that spans time periods 1810–1860 (*C*_1_) and 1960–2010 (*C*_2_) respectively, and there are 37 target word types (33 nouns and 4 verbs) for evaluation.

We construct an inf-mix model for predicting semantic change in the following way: for each target word *w*, we first use a deep contextualized neural language model named BERT (Devlin et al., 2019) to encode each usage sentence of *w* into a high-dimensional sentence embedding space. We then run the DP-Means clustering algorithm on two sets of usage sentence embeddings in *C*_1_, *C*_2_ respectively to obtain two groups of induced centroid embeddings $H_{w}^{\left(1\right)},H_{w}^{\left(2\right)}$. The degree of semantic change of *w* between the two time periods of study can then be quantified as the mean pairwise cosine distance between $H_{w}^{\left(1\right)},H_{w}^{\left(2\right)}$:

$$s_{w}\left(t_{1},t_{2}\right)=\frac{1}{|H_{w}^{\left(1\right)}|\cdot |H_{w}^{\left(2\right)}|}\sum_{h_{1}\in H_{w}^{\left(1\right)},h_{2}\in H_{w}^{\left(2\right)}}\frac{\langle h_{1},h_{2}\rangle }{∥h_{1}{∥}^{2}∥h_{2}{∥}^{2}}$$ (9)

We evaluate the models by computing the Spearman’s *ρ* correlation score between predicted semantic change scores and the gold-standard results by human annotators. Similar to the previous three case studies, we varied the trade-off parameter *λ* of the inf-mix model from 0 up to a sufficiently large value of 0.7 with a step size of 0.05, and take the one with highest silhouette clustering score as the optimal inf-mix model. The case of *λ* = 0 and *λ* = 0.7 again correspond to a prototype-based and an exemplar-based LSC model and can be taken as two baselines.

**Figure 11.**
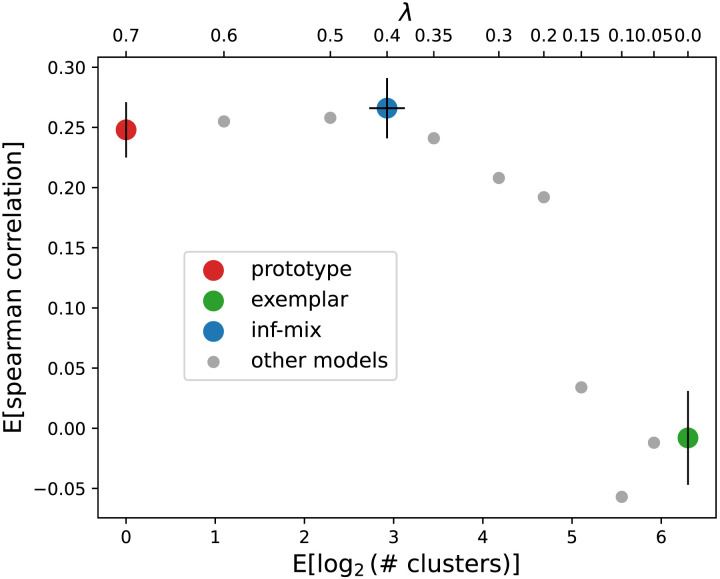
Correlations between inf-mix model predictions and human annotated degree of semantic change for 37 English words between two historical time periods. The gray dots show the spectrum of infinite mixture models under different *λ* values and varying numbers of inferred semantic centroids (memory complexity). The left end (red dot) of the x-axis corresponds to the prototype model with *λ* ≥ 0.7. The right end (green dot) of the x-axis corresponds to the exemplar model with *λ* = 0. The blue dot corresponds to the infinite mixture model with inferred optimal *λ* = 0.15.

Figure 11 shows correlation scores for a series of inf-mix models. We observe that the inf-mix model with highest clustering quality (measured by silhouette score) coincides with the inf-mix model (*λ* = 0.15) of highest correlation between human LSC judgements (*ρ* = 0.266, *p* < 10^−11^) compared to the prototype (*ρ* = 0.245, *p* < 10^−7^) and the exemplar (*ρ* = −0.013, *p* < 10^−6^) models. Notably, the exemplar model that yields best predictive accuracy in the previous three case studies now performs much worse compared to models of lower memory costs, suggesting that semantic abstraction plays a more essential role than individual usage memorization in modeling diachronic lexical semantic change. On the other hand, the inf-mix model yields either the best or a near-optimal performance across all four studies, suggesting the important role of cognitive efficiency in explaining word meaning change over time. Table 2 shows several example lemmas on which the inf-mix model yields the best predictive accuracy in LSC. Though not perfect, we found that the inf-mix model is better than the alternative models in capturing prominent word meaning changes (e.g. *tip*), and words with relatively stable meaning (e.g. *chairman*).

**Table 2.** Sample English lemmas in SemEval-2020 Task 1 with ground-truth and model predicted scores of lexical semantic change (LSC) between the time periods of 1810–1860 and 1960–2010.

Lemma	Ground-truth LSC	Prototype predicted LSC	Exemplar predicted LSC	Inf-mix predicted LSC
chairman (noun)	0	−0.012	0.133	0.008
player (noun)	0.274	0.557	0.182	0.306
tip (verb)	0.679	−0.197	−0.281	0.223

## DISCUSSION AND CONCLUSION

We presented an efficiency-based computational framework of semantic chaining for modeling the historical extension of word meaning. Our framework provides a synthesis of existing approaches and suggests that different forms of chaining can be understood as a trade-off between model representational accuracy and memory complexity. Our study moves away from the typical focus on a comparative analysis of different chaining models but instead develops a unified view toward interpreting the diverse kinds of chaining in word meaning extension. In particular, we showed that the most commonly described chaining models based on the exemplar and prototype theories can be interpreted as two extremes of a trade-off for cognitive efficiency.

Our work extends beyond existing work on chaining by proposing a new class of models that supports flexible growth of meaning clusters based on historical and emerging word usages. We showed that the infinite mixture chaining model is on par with the exemplar model and performs better than the prototype model in reconstructing the historically emerged noun-argument pairings with English verbs and adjectives, and numeral classifiers in Mandarin Chinese. We also showed that the same model yields a substantially more compact representation for the internal structures of word meaning compared to the exemplar model, and therefore near-optimally trading off model accuracy with complexity.

Our framework has several limitations. Firstly, we considered a simplified problem of word meaning extension by predicting how words should pair up with previously unattested concepts as they emerged over time. However, word meaning may change without involving novel compositions. For instance, the phrase *to save my key* used to refer to “keeping a physical key”, but the same phrase took on the novel meaning “to save a string that gives access to retrieve information” without requiring any change in its compositional form. Additionally, word meaning extension may occur in a highly contextual setting that is not necessarily reflected in novel argument pairing. For example, saying “that person is sick” can be interpreted as a sick person or a cool person, depending on the context of the communicative scenario. Our emphasis on predicting emerging argument and argument pairing is motivated by a comparison to existing approaches to modeling semantic chaining which use a similar setup for prediction (Grewal & Xu, 2021; Habibi et al., 2020; Xu et al., 2016; Yu & Xu, 2021), and it can be taken as an initial step toward characterizing the general processes of word meaning extension.

Secondly, recent work has shown that chaining models based on semantic proximity between novel and existing meaning (also known as “associative chaining”) often fail to predict metaphorical or other non-literal word meaning extensions (e.g., “to arrive at school” → “to arrive at conclusion”) (Yu, 2023). Future work should explore how infinite mixture models may be integrated with other types of chaining mechanisms to account for mechanisms such as analogy (Fugikawa et al., 2023), which is also discussed in structural mapping (Gentner, 1983) and conceptual metaphor mapping (Lakoff & Johnson, 2008).

Thirdly, our study focused on modeling the cognitive mechanisms that may give rise to novel word choices in lexical evolution, but it does not account for other mechanisms or factors that can also shape the changing landscapes of the lexicon. For example, novel word meanings might emerge due to growing needs for communicating socio-cultural changes or technological innovations, and the chaining models we presented here do not take into account these factors. Related to this issue, we evaluated our framework against historical corpus data that might reflect conventionalized or sustained changes, but this approach is potentially limited in explaining spontaneous or less common changes that do not appear in text corpora. Understanding rare, unconventional patterns of word meaning extension may require a diachronic analysis of linguistic communities, and a characterization of the elimination and propagation of linguistic innovations through the lens of sociolinguistics.

Finally, the current framework of infinite mixture chaining considers word meaning extension at a population level by predicting historically emerging word usages presumably shared among a group of language speakers. We believe that our framework has the potential to explain word meaning extension from individuals. Prior studies have shown that computational models of prototype-based and exemplar-based chaining can explain novel word uses by (individual) children (Pinto & Xu, 2021; Xu & Xu, 2021), and future work can investigate whether infinite mixture chaining might offer an individual-level account of word meaning extension in light of cognitive efficiency.

We have developed a general probabilistic framework for reconstructing emerging word meanings through time and explored a broad class of chaining models that trade off representational accuracy with memory complexity. Our work provides a unified account for the different forms of chaining grounded in that rational models of human and machine learning, and it also opens the avenue for exploring the cognitive efficiency of word meaning acquisition and representation in the mind.

## ACKNOWLEDGMENTS

We thank Suzanne Stevenson, the editor, and the anonymous reviewers for their feedback on our work.

## FUNDING INFORMATION

This work was supported by an NSERC Discovery Grant RGPIN-2018-05872, a SSHRC Insight Grant 435190272, and an Ontario ERA Award #ER19-15-050 to YX.

## AUTHOR CONTRIBUTIONS

LY and YX conceptualized and designed the study, and developed the models. LY acquired data, implemented the models and performed the analyses. LY and YX interpreted the results and wrote the paper. YX acquired funding.

## DATA AVAILABILITY STATEMENT

All datasets we used in this study are publicly available and can be downloaded via the following links: Google Syntactic n-gram: https://commondatastorage.googleapis.com/books/syntactic-ngrams/index.html; SemEval 2020 Task 1: https://competitions.codalab.org/competitions/20948; Historical data of Chinese numeral classifiers: https://github.com/AmirAhmadHabibi/ChainingClassifiers.

## Notes

^1^ Precisely, the inf-mix model will become the prototype model as long as *λ* is greater than the maximum pairwise Euclidean distance between any two support noun embeddings.^2^ We took the averaged Silhouette score over clustering of all support sets across all decades, and found that the inf-mix model with *λ* = 0.24 yields the highest mean Silhouette score.

## APPENDIX: RESULTS OF NOUN ARGUMENT PREDICTION PROBLEMS

Additionally to the task of head prediction described in the main text, we also assess the efficiency of different chaining models on the task of predicting novel noun arguments. Figure A1 summarizes the results for noun argument prediction of English head verbs in Case Study 1. We observe that similar to the head prediction problems, among all the models examined, the infinite mixture and exemplar models yielded near-equivalent predictive accuracy and are superior than the alternatives. The prototype and 1nn models perform better than the two baselines, but they are much worse than the top 2 models. Similar trends are also observed in the noun argument prediction problems of English adjectives (Figure A3) and Chinese numeral classifiers (Figure A5).

Figure A2 shows the efficiency trade-off result of noun argument prediction for English verbs in Case Study 1. Again, similar to the trade-off results in head prediction problem, we found that 1) by sweeping *λ* from 0 toward ∞ (in this case *λ* ≥ 0.5 suffices), the predictive accuracy drops only slightly from the exemplar model to the infinite mixture model (*λ* = 0.24) but substantially to the prototype model—this finding confirms with our previous analysis, that the infinite mixture model predicts on par with the exemplar model; and 2) the marginal gain on accuracy of the exemplar model comes at a high cost in memory complexity: compared to the infinite-mixture model, it requires over 2^5^-fold more storage of cluster centroids to achieve a gain of <0.01 in predictive accuracy. We observed a similar trade-off curve for the argument noun prediction problems of English adjectives (Figure A4) and Chinese numeral classifiers (Figure A6) as well. Overall, the infinite mixture model achieves a better balance between precision and memory.
